# MicroRNA‐378a‐3p contributes to ovarian cancer progression through downregulating PDIA4

**DOI:** 10.1002/iid3.350

**Published:** 2020-11-07

**Authors:** Yao Chanjiao, Chen Chunyan, Qiu Xiaoxin, Han Youjian

**Affiliations:** ^1^ No. 3 Department of Obstetrics and Gynecology Hunan Provincial People's Hospital Changsha China; ^2^ Department of cardiology Hunan Provincial People's Hospital Changsha China

**Keywords:** invasion, microRNA‐378a‐3p, migration, ovarian cancer, PDIA4, PI3K/AKT signaling pathway, proliferation

## Abstract

**Objective:**

MicroRNAs, as essential players in tumorigenesis, have been demonstrated to have a revolutionary effect on human cancer research. Ovarian cancer is the primary reason of death among gynecologic malignancies. In view of this, it is significant to identify prognostic and predictive markers for treatment of ovarian cancer. The aim of this study was to probe into the effects of miR‐378a‐3p and protein disulfide‐isomerase A4 (PDIA4) on the biological functions of ovarian cancer cells.

**Methods:**

miR‐378a‐3p expression and PDIA4 messenger RNA expression in human ovarian cancer cells, normal human ovarian epithelial cells, and serum of both ovarian cancer patients and healthy people were detected by reverse transcription‐quantitative polymerase chain reaction, and the PDIA4 protein expression was tested by Western blot analysis. Ovarian cancer OVCAR3 and SKOV3 cells were transfected or cotransfected with miR‐378a‐3p mimic or pcDNA3.1‐PDIA4 or their negative control plasmids to explore their roles in biological functions in ovarian cancer cells. Luciferase activity and RIPA assays were implemented to validate the interaction between miR‐378a‐3p and PDIA4. Western blot analysis was utilized to detect phosphatidylinositol‐3 kinase/serine/threonine kinase (PI3K/AKT) signaling pathway‐related protein expression and their phosphate expression levels.

**Results:**

miR‐378a‐3p was elevated and PDIA4 was decreased in ovarian cancer cells and serum. In addition, miR‐378a‐3p mimic induced ovarian cancer cell growth, while miR‐378a‐3p inhibitor and pcDNA3.1‐PDIA4 presented an inverse trend. pcDNA3.1‐PDIA4 partially eliminated the capabilities of miR‐378a‐3p mimic on ovarian cancer progression. Meanwhile, miR‐378a‐3p was found to negatively regulate PDIA4, and miR‐378a‐3p mimic increased the phosphorylation levels of AKT and PI3K, while pcDNA3.1‐PDIA4 exhibited an opposite tendency. Furthermore, pcDNA3.1‐PDIA4 largely eliminated the functions of miR‐378a‐3p mimic on phosphorylation levels of AKT and PI3K.

**Conclusion:**

This study provides evidences that miR‐378a‐3p activates PI3K/AKT signaling pathway by modulating PDIA4 expression, thereby playing a role in promoting the growth of ovarian cancer cells. This study provides novel directions for targeted therapy of ovarian cancer.

## Graphical abstract

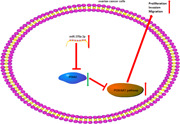

## 1 INTRODUCTION

Ovarian cancer has become one of the leading causes of gynecological cancer‐related deaths with high mortality in women.^1^ Knowingly, several histotypes of ovarian cancer have been recognized, each with specific genomic and epidemiologic characteristics.^2^ Epithelial ovarian cancer, arising from the epithelium cells of the ovary, fallopian tube, and peritoneum, accounts for approximately 90% of ovarian cancer, while the remaining 10% of ovarian tumors originate from gonadal stromal cells and germ cells.^3^ In recent years, much progress has been made in screening and prevention of ovarian cancer. Improved tools which combine both genetic and epidemiologic factors for the prediction of a person's ovarian cancer risk are used to develop preventive and screening methods.^4^ Nevertheless, reliable diagnostic indicators and approaches for early detection and screening of ovarian cancer are still lacking. Thus, identifying the useful clinical markers would benefit the treatment of ovarian cancer.

MicroRNAs (miRNAs) have either oncogenic or tumor suppressor functions. The upregulated miRNAs act as oncogenes through downregulating tumor‐inhibiting genes, but the downregulated miRNAs function as tumor suppressor genes via negatively modulating oncogenes.^5^ Numerous studies have suggested that dysregulation of miRNAs could function in ovarian cancer, and these miRNAs would be applied in treating ovarian cancer.^5^, ^6^ Evidence has shown that miR‐378 is expressed in many cancer cells.^7^, ^8^ In addition, a large number of articles have demonstrated that miR‐378a‐3p plays a role in various types of cancers, including glioblastoma multiforme, esophageal squamous cell carcinoma, and colorectal cancer.^9^, ^10^, ^11^ It is also reported that miR‐378 or miR‐378a‐3p is implicated in ovarian cancer development.^12^, ^13^ miRNAs are revealed to be extensively involved in the promotion and suppression of cancers by controlling thousands of cancer‐related genes.^14^ In this current research, the online website predicted the bindings site between miR‐378a‐3p and protein disulfide‐isomerase A4 (PDIA4). Protein disulfide isomerase (PDI) family members, such as PDIA4, are demonstrated to exert functions in the pathogenesis of numerous diseases.^15^ Evidence has shown that that PDIA4 is lowly expressed in platinum‐resistant ovarian cancer patients.^16^ A recent study has pointed out that PDIA4 might be considered as therapeutic biomarkers for managing ovarian cancer.^17^ The known oncogenic pathways have provided important insights into the capabilities of miRNAs in human cancers.^6^ A recent article has indicated that the phosphatidylinositol‐3 kinase/serine/threonine kinase (PI3K/AKT) pathway is closely related to ovarian cancer.^18^ In addition, a large number of studies have shown that the PI3K/AKT signaling pathway plays an important role in the invasion and migration of ovarian cancer.^19^, ^20^ Rückerl et al.^21^ have identified the potential miR‐378a‐3p targets in the PI3K/AKT pathway.^21^ Therefore, we have generated a miR‐378a‐3p expression construct to identify the biological functions of miR‐378a‐3p. Here, we show that miR‐378a‐3p plays a role in malignant phenotypes of ovarian cancer cells.

## 2 MATERIALS AND METHODS

### 2.1 Ethical approval

This study was approved by the ethics committee of the Hunan Provincial People's Hospital, and the experimental procedures were in line with the Declaration of Helsinki. Each patient signed an informed consent before the collection and usage of clinical samples.

### 2.2 Study subject and sample collection

Forty‐three clinical serum samples were collected from ovarian cancer patients (aged between 28 and 56 years, with a median age of 39 years) in Hunan Provincial People's Hospital between 2017 and 2018. Meanwhile, eight control serum samples were harvested from the healthy controls (aged 24–49 years old, with a median age of 41 years) who were examined in Hunan Provincial People's Hospital from 2017 to 2018.

### 2.3 Cell culture and transfection

Cells were incubated for 24 h at 37°C in Roswell Park Memorial Institute‐1640 medium with 10% (vol/vol) fetal calf serum (Invitrogen; Thermo Fisher Scientific, Inc.) in a humidified chamber with 5% CO_2_. Human ovarian epithelial cell line (HOSE) and ovarian cancer cells (OVCAR3 [adenocarcinoma], CaOV3 [adenocarcinoma], SKOV3 [adenocarcinoma], and OV90 [malignant papillary serous adenocarcinoma]) were available from American Type Culture Collection. SKOV3 and OVCAR3 cell lines grown in the logarithmic phase were transfected with 100 nMmiR‐378a‐3p mimic, miR‐378a‐3p inhibitor, miR‐378a‐3p mimic negative control (named as mimic NC), miR‐378a‐3p inhibitor NC (named as inhibitor NC), 2 μg pcDNA3.1 or 2 μg pcDNA3.1‐PDIA4 (all from RiboBio Co., Ltd.). Cell transfection was implemented with the application of Lipofectamine 2000 transfection reagent (Invitrogen) referring to the related instructions. The transfected cells were cultured with serum‐free medium and then renewed with medium supplemented with 10% fetal bovine serum (FBS) 8 h later. The cells were placed for 48 h at 37°C in a constant temperature incubator with 5% CO_2_ and 95% humidity, and RNA was collected and extracted. The extracted protein was implemented for subsequent experiments.

### 2.4 Reverse transcription‐quantitative polymerase chain reaction

SKOV3 and OVCAR3 cells were dissolved in 1 ml TRIzol (Thermo Fisher Scientific), and total RNA was extracted by the requirements of TRIzol kit and then reversely transcribed into complementary DNA. The PCR reaction system was configured in the light of the instructions of the fluorescence quantitative PCR (Takara) kit. Real‐time quantitative PCR was launched by ABI 7500 quantitative PCR instrument (Applied Biosystems). Primer sequences were presented in Table 1 and synthesized by Genewiz Biotechnology. U6 was an internal control for miR‐378a‐3p while glyceraldehyde‐3‐phosphate dehydrogenase for PDIA4. 2^‐ΔΔCt^ Method was utilized for data assessment.^22^

**Table 1:** Primer sequence

Name of primer	Sequences (5’–3’)
PDIA4	F: CCTGCAGAAATTAGAACGCGG
	R: CCACCAGCTTTGTAACCAGTC
miR‐378a‐3p	F: ACACTCCAGCTGGGTTTGGCAATGGTAGAACT
	R: TGGTGTCGTGGAGTCG
GAPDH	F: ACCACAGTCCATGCCATCAC
	R: TCCACCACCCTGTTGCTGTA
U6	F: TCGCTTCGGCAGCACATATAC
	R: GCGTGTCATCCTTGCGCAG

Abbreviations: F, forward; GAPDH, glyceraldehyde phosphate dehydrogenase; GREB1, growth regulation by estrogen in breast cancer 1; miR‐378a‐3p, microRNA‐378a‐3p; R, reverse.

### 2.5 3‐(4,5‐Dimethylthiazol‐2‐yl)‐2,5 diphenyltetrazolium bromide assay

The ovarian cancer cells in the logarithmic phase were transfected with the corresponding plasmids, and cell count was performed after 24, 48, 72, and 96 h of transfection. The cell suspension (100 μl, equal to 10^4^–10^5^ cells) was seeded in a 96‐well cell culture plate. Each cell was provided with three parallel wells, and cells were then cultured in a 37°C, 5% CO_2_ cell incubator. Afterward, each well was appended with 20 μl 3‐(4,5‐dimethylthiazol‐2‐yl)‐2,5 diphenyltetrazolium bromide (MTT) solution (5 mg/ml; Sigma‐Aldrich Chemical Company), and cells were continued to be incubated for 4 h. After discarding the culture solution, each well was supplemented with 150 μl dimethyl sulphoxide for crystal dissolution. The optical density (OD) value at the wavelength of 495 nm (OD_495_ value) of each well was measured with the application of a microplate reader. MTT curve was drawn with the OD value as the ordinate and the time as the abscissa.

### 2.6 Transwell invasion assay

After the Matrigel‐coated chamber was taken out from −20°C and melted at room temperature, 0.5 ml of serum‐free culture solution was appended to the Transwell chamber (Coring) and a 24‐well culture plate for 2‐h culture. Cells (1 × 10^5^) were seeded into the corresponding Transwell chamber and supplemented with culture solution containing 1% FBS, while the outer layer culture well was appended with 0.75 ml culture solution containing 15% FBS. With 20‐h incubation, the Transwell chamber was rinsed with phosphate‐buffered saline (PBS), fixed by 4% paraformaldehyde for 10 min and dyed by crystal violet solution for 10 min. Next, the upper Matrigel and cells were gently wiped off with cotton swabs. Five fields of view were randomly photographed and cells in each field of view were counted. The mean and *SD* were calculated for the statistical analysis.

### 2.7 Scratch test

Cell scratch test was conducted as previously described.^23^ In brief, the cells of the control and the experimental groups were spread on a six‐well plate. When growing to 90% confluence, three straight lines were drawn in the plate with a 100 μl pipette tip. Subsequently, the floating cells were renewed with serum‐free medium, and next, cultured with or without exosomes. The empty lanes between the cells were observed with a low power phase‐contrast microscope (Olympus MK). Furthermore, the gap changes in the blank space were viewed after 24 h of continuous cultivation, and the increased area was calculated by ImageJ.

### 2.8 Dual‐luciferase reporter gene assay

Biological prediction software Target Scan (http://www.targetscan.org/vert_72) and Starbase (http://starbase.sysu.edu.cn/) was utilized for prediction of the potential binding site of miR‐378a‐3p and PDIA4. In the light of the prediction results, the mutant and wild sequences of PDIA4 and miR‐378a‐3p binding sites were designed, respectively, which were then inserted into the luciferase reporter gene vector (pGL3‐Basic), named as mutant type (MT)‐PDIA4 and wild‐type (WT)‐PDIA4, respectively. MT‐PDIA4 or WT‐PDIA4 and miR‐378a‐3p mimic or miR‐378a‐3p inhibitor were cotransfected into HEK‐293T cells, and a luciferase dual reporter gene kit (Promega) was implemented to detect Firefly luciferase activity and Renilla luciferase activity of each group 48 h posttransfection. Renilla luciferase activity was utilized as an internal reference, and the ratio of Firefly luciferase activity to Renilla luciferase activity was the relative luciferase activity.

### 2.9 Coimmunoprecipitation

miR‐378a‐3p mimics‐transfected SKOV3 and OVCAR3 cells were washed three times with 4°C PBS, then added with radioimmunoprecipitation assay (RIPA) buffer (Thermo Fisher Scientific) containing protease inhibitors, lysed at 4°C for 30 min and centrifuged at 12,000*g* for 15 min. The protein supernatant was obtained to measure the protein concentration. After that, 200 μg total protein extracted solution was diluted to a concentration of 2 μg/μl with RIPA buffer containing protease inhibitor (Merck), and then added with 10 μl anti‐PDIA4. The corresponding volume of anti‐immunoglobulin G (IgG; Invitrogen) was appended in the NC group. Afterward, the clean 30 μl Protein G Agrose was supplemented to the protein sample after antibody incubation overnight and centrifuged at 14,000*g* for 1 min to remove the supernatant. Next, with RIPA buffer washing for three to five times, 30 μL 2X Laemmli sample buffer was applied for dissolving protein. Last, the protein was heated at 100°C for 5 min and centrifuged to remove the supernatant, and then the immunoprecipitation was detected by Western blot analysis.

### 2.10 IP‐RT‐PCR

The Protein‐RNA complex obtained from the IP was appended to 200 μl RIPA buffer to resuspend the bead protein complex, incubated at 70°C for 45 min in a metal bath, and supplemented with 500 μl TRIzol (Invitrogen) for RNA extraction. The obtained RNA was used for subsequent PCR detection.

### 2.11 Western blot assay

Cells were rinsed three times with prechilled PBS, and then lysed with protein extraction lysate (100 μl/50 ml culture flask) and placed on ice for 30 min. After protein centrifugation at 12,000 rpm for 10 min, the obtained supernatant was subpacked in 0.5 ml centrifuge tubes and store at −20°C or bicinchoninic acid kit for protein quantification. The extracted protein was supplemented with the 6X sodium dodecyl sulfate loading buffer, followed by 10% polyacrylamide gel electrophoresis protein separation. Electroblotted onto a polyvinylidene fluoride membrane, the protein was blocked with 5% skim milk powder, probed with primary antibody against PDIA4 (1:1000; ab155800), β‐actin (1:5000; ab8277; both from Abcam), PI3K/AKT pathway‐related protein PI3K (1:1000; 4255S), phosphorylated (p)‐PI3K (1:1000; 17366S), AKT (1:1000; 4691S) and p‐AKT (1:1000; 13038S; all from Cell Signaling Technology), and next, reprobed with the secondary antibody, goat anti‐rabbit IgG (1:5000; Beijing ComWin Biotech Co., Ltd.). After developing, the expression levels of these proteins were detected. With β‐actin as an internal reference, ImageJ was utilized to calculate the gray value of each protein band.

### 2.12 Statistical analysis

SPSS 18.0 (IBM Corp) and GraphPad Prism 6.0 (GraphPad Software Inc) were utilized for the data process. The measurement data were depicted as mean ± *SD*. The comparison between the two groups was analyzed by *t* test, and the comparison among multiple groups was analyzed by one‐way analysis of variance. Linear correlation analysis was evaluated with the use of the Pearson correlation coefficient analysis. The survival analysis of miR‐378a was conducted by online software Kaplan–Meier Plotter (https://kmplot.com/analysis/index.php?p=service), and the survival analysis of PDIA4 was conducted by the GEPIA database (http://gepia.cancer‐pku. cn/detail. php?). *p* < .05 was indicative of significant difference.

## 3 RESULTS

### 3.1 High miR‐378a‐3p expression and low PDIA4 expression are found in serum of ovarian cancer patients

**Figure 1:**
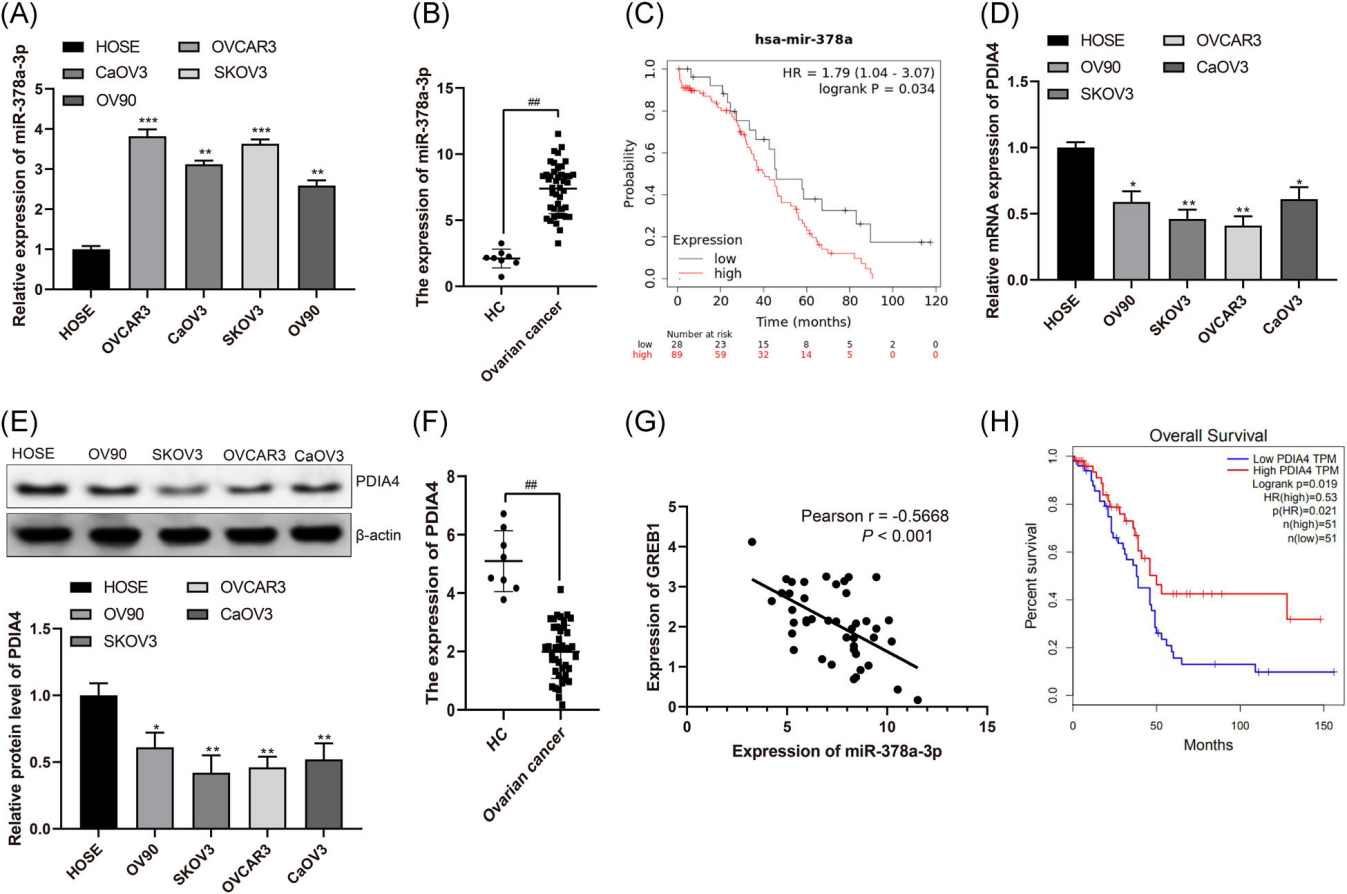
miR‐378a‐3p is highly expressed and PDIA4 is poorly expressed in the serum of ovarian cancer patients and ovarian cancer cells. A, RT‐qPCR was performed to determine miR‐378a‐3p expression in normal human ovarian epithelial cells (HOSE) and ovarian cancer cells (OVCAR3, CaOV3, SKOV3, and OV90). B, RT‐qPCR was conducted to determine miR‐378a‐3p expression in serum of ovarian cancer patients and healthy volunteers. C., Survival analysis of miR‐378a‐3p by Kaplan–Meier Plotter online software. D and E, RT‐qPCR and Western blot assay were utilized to detect PDIA4 mRNA and protein expression in normal HOSE and ovarian cancer cells (CaOV3, SKOV3, OVCAR3, and OV90). F, RT‐qPCR was conducted to determine PDIA4 mRNA expression in serum of ovarian cancer patients and healthy volunteers. G, Pearson correlation analysis was performed for the correlation between miR‐378a‐3p and PDIA4. H, TCGA database was implemented to analyze the survival of PDIA4 in ovarian cancer. All data were expressed as mean ± *SD* (*n* = 3). *p < .05, **p < .01, ***p < .001 versus HOSE group, ^##^ *p* < .01 versus HC group. mRNA, messenger RNA; RT‐qPCR, reverse transcription‐quantitative polymerase chain reaction; TCGA, The Cancer Genome Atlas

As previously reported, miR‐378a‐3p is upregulated in ovarian cancer cells, and we found that miR‐378a‐3p expression was in high expression in ovarian cancer cells (OVCAR3, CaOV3, SKOV3, and OV90) versus normal HOSE (Figure 1A, *p* < .01). In addition, through the detection of clinical serum samples, it was found that miR‐378a‐3p expression was elevated in serum of patients with ovarian cancer in contrast to healthy people (Figure 1B, *p* < .01). Meanwhile, the survival analysis of miR‐378a‐3p by online software Kaplan–Meier Plotter (https://kmplot.com/analysis/index.php?p=service) showed that miR‐378a‐3p was highly expressed and had a poor prognosis. Low expression of miR‐378a‐3p reflected better prognosis (Figure 1C, *p* < .01). Similarly, our study found that PDIA4 expression was reduced in ovarian cancer cells versus that of HOSE cells (Figure 1D,E, *p* < .01). At the same time, serum level of PDIA4 was declined in ovarian cancer patients in contrast to healthy people (Figure 1F, *p* < .01). Subsequently, correlation analysis found that miR‐378a‐3p was negatively correlated with PDIA4 (Figure 1G, *p* < .01). Moreover, the survival analysis of the The Cancer Genome Atlas (TCGA) database showed that low expression of DIA4 indicated poor prognosis, while high expression of which presented an inverse trend (Figure 1H, *p* < .01). These results imply that miR‐378a‐3p and PDIA4 are of significance in ovarian cancer development, and SKOV3 and OVCAR3 cells were screened for subsequent experiments with respect to the miR‐378a‐3p and PDIA4 expression in cells.

### 3.2 miR‐378a‐3p promotes ovarian cancer cell invasion and migration

**Figure 2:**
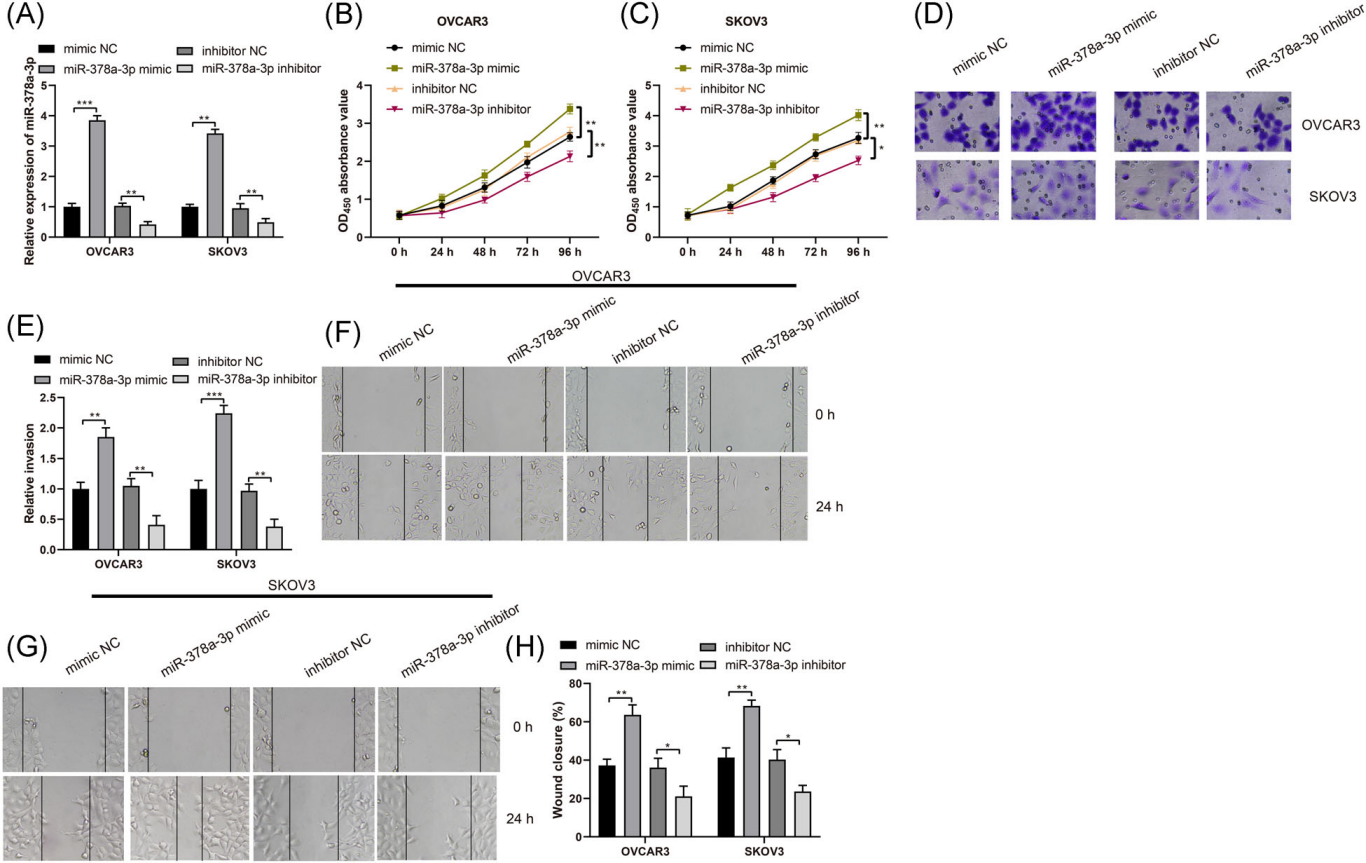
miR‐378a‐3p induces ovarian cancer cell invasion and migration. Ovarian cancer cells OVCAR3 and SKOV3 were transfected with miR‐378a‐3p mimic, miR‐378a‐3p inhibitor or negative control, respectively. A, miR‐378a‐3p expression was determined by RT‐qPCR. B and C, Viability of OVCAR3 and SKOV3 cells was measured by MTT assay. D and E, Invasion ability of OVCAR3 and SKOV3 cells was detected by Transwell assay. F and H, Migration ability of OVCAR3 and SKOV3 cells was detected by scratch test. All data were expressed as mean ± *SD* (*n* = 3). *p < .05, **p < .01, ***p < .001. MTT, 3‐(4,5‐dimethylthiazol‐2‐yl)‐2,5 diphenyltetrazolium bromide; RT‐qPCR, reverse transcription‐quantitative polymerase chain reaction

Since miR‐378a‐3p expression in OVCAR3 and SKOV3 cells (two most common highly metastatic cell lines in ovarian cancer) was found to be higher in comparison with other ovarian cancer cells, we selected these two kinds of cells lines to elucidate whether miR‐378a‐3p affected the invasion and migration abilities of ovarian cancer cells. We transfected miR‐378a‐3p mimic or miR‐378a‐3p inhibitor or their respective NCs into OVCAR3 and SKOV3 cells, the results of which indicated that miR‐378a‐3p mimic enhanced miR‐378a‐3p expression while miR‐378a‐3p inhibitor declined miR‐378a‐3p expression (Figure 2A, *p* < .01), implying miR‐378a‐3p had great transfection efficiency in OVCAR3 and SKOV3 cells, which could be used in subsequent experiments. Meanwhile, findings also showed that miR‐378a‐3p mimic trended toward a promotion in cell proliferation, migration, and invasion, while miR‐378a‐3p inhibitor functioned inversely (Figure 2B–H, all *p* < .01). To conclude, this indicates that miR‐378a‐3p promotes ovarian cancer cell growth and miR‐378a‐3p inhibitor suppresses cell growth.

### 3.3 PDIA4 restricts ovarian cancer cell invasion and migration

**Figure 3:**
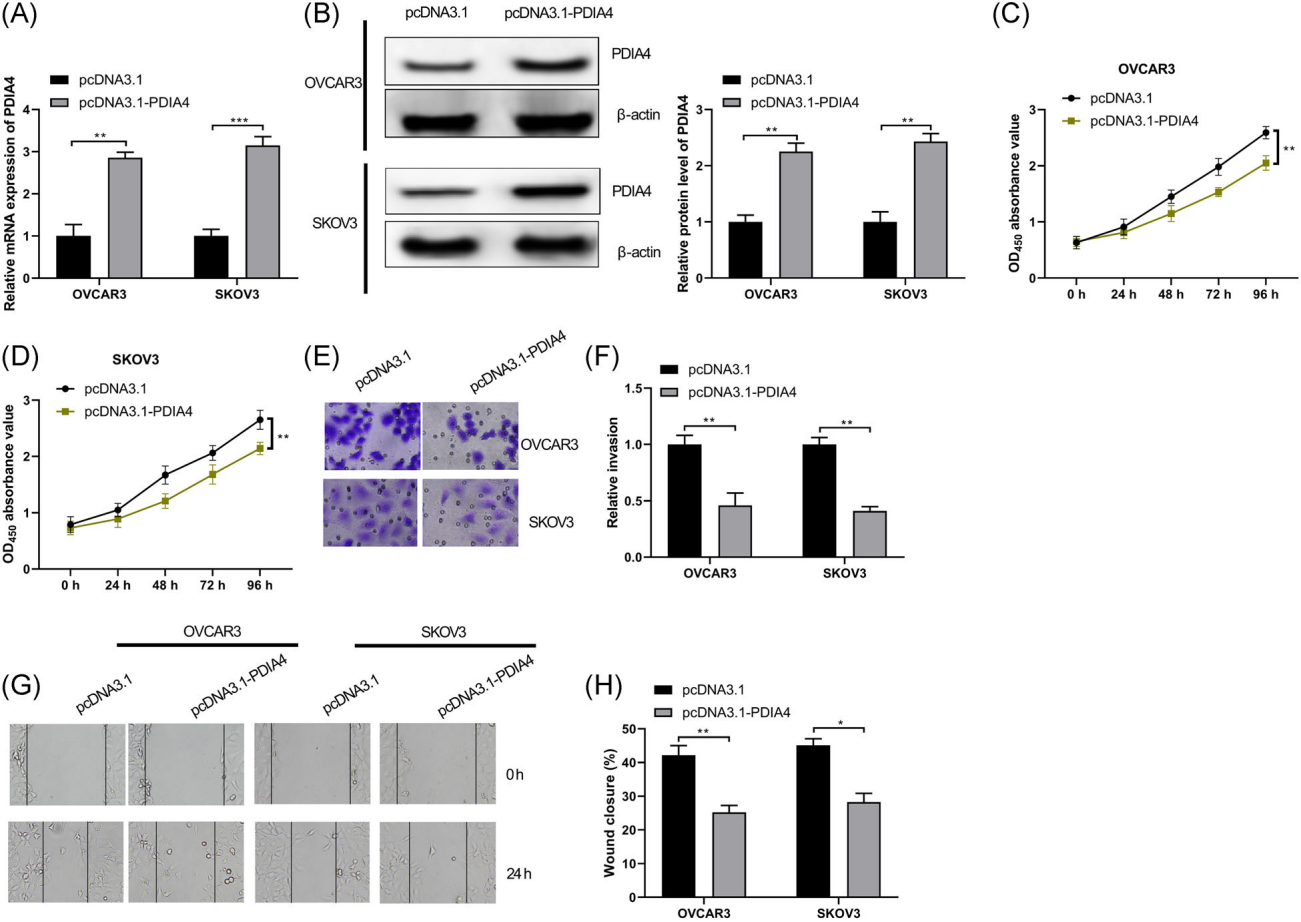
PDIA4 blocks ovarian cancer cell invasion and migration. Ovarian cancer cells OVCAR3 and SKOV3 were transfected withpcDNA3.1 or pcDNA3.1‐PDIA4, respectively. A, PDIA4 mRNA expression was determined by RT‐qPCR. B, PDIA4 protein expression in OVCAR3 and SKOV3 cells was detected by Western blot assay. C and D, Viability of OVCAR3 and SKOV3 cells was measured by MTT assay. E and F, Invasion ability of OVCAR3 and SKOV3 cells was detected by Transwell assay. G and H, Migration ability of OVCAR3 and SKOV3 cells was detected by scratch test. All data were expressed as mean ± *SD* (*n* = 3). *p < .05, **p < .01, ***p < .001. mRNA, messenger RNA; MTT, 3‐(4,5‐dimethylthiazol‐2‐yl)‐2,5 diphenyltetrazolium bromide; RT‐qPCR, reverse transcription‐quantitative polymerase chain reaction

Next, we then verified the effect of PDIA4 on the growth of ovarian cancer cells. Considering the downregulated expression of PDIA4 in ovarian cancer as previously reported, we only transfected the overexpressed PDIA4 plasmid pcDNA3.1‐PDIA4 or control plasmid pcDNA3.1 into OVCAR3 and SKOV3 cells. Based on the obtained results, we found that upregulation of PDIA4 elevated PDIA4 expression (Figure 3A,B, *p* < .01), suggesting that PDIA4 was well expressed in OVCAR3 and SKOV3 cells and could be used in subsequent experiments. Meanwhile, upregulated PDIA4 trended toward a decrease in cell proliferation, migration, and invasion (Figure 3C–H, all *p* < .01), implying that PDIA4 plays a part in the suppression of ovarian cancer cell growth.

### 3.4 miR‐378a‐3p targets PDIA4

**Figure 4:**
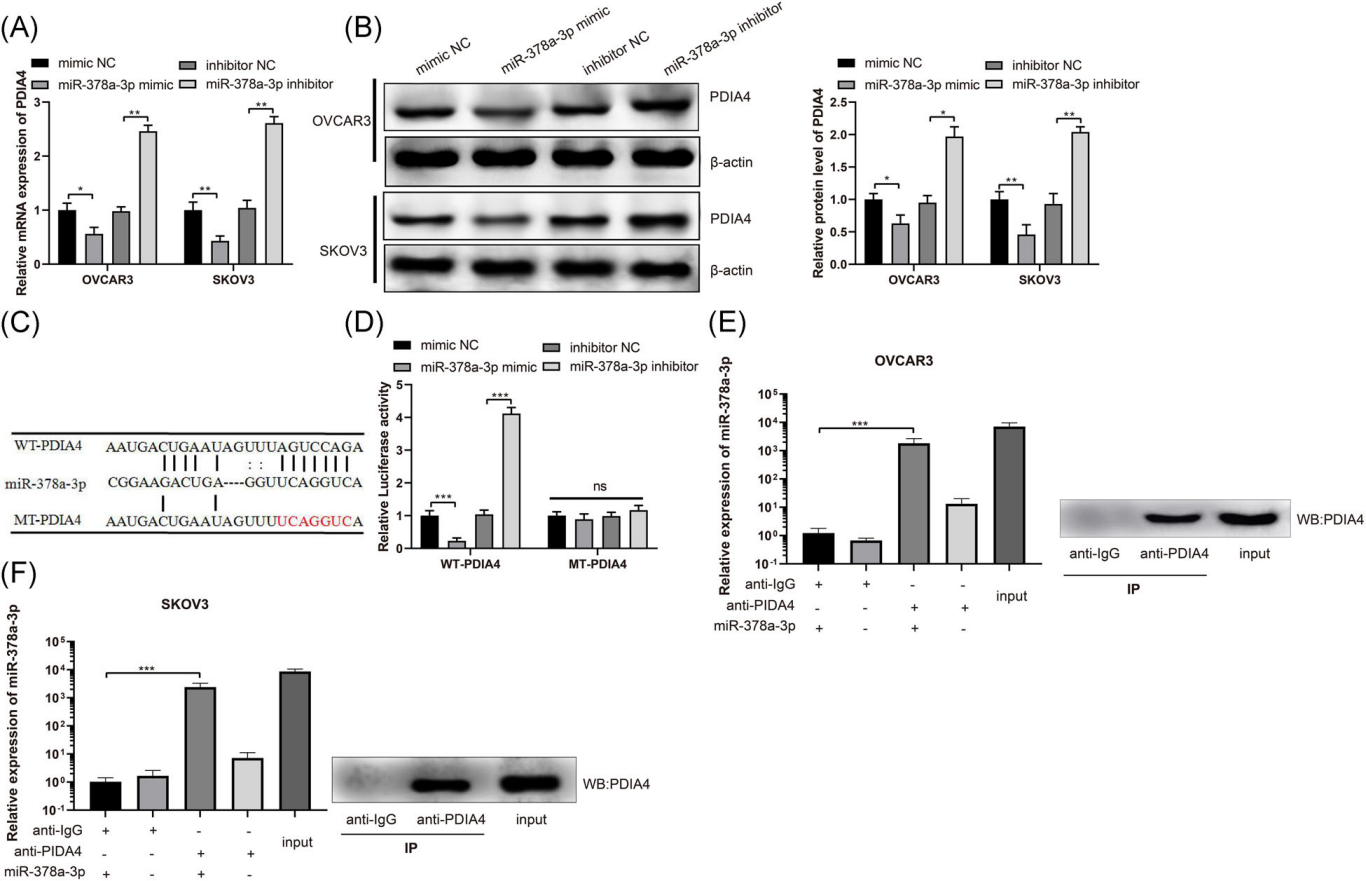
miR‐378a‐3p negatively regulates PDIA4. Ovarian cancer cells OVCAR3 and SKOV3 were transfected with miR‐378a‐3p mimic, miR‐378a‐3p inhibitor or negative control, respectively. A, PDIA4 mRNA expression in OVCAR3 and SKOV3 cells was determined by RT‐qPCR. B, PDIA4 protein expression was detected by Western blot assay. C, The online software Starbase was searched to predict the targeting sites of miR‐378a‐3p and PDIA4 and the designed PDIA4 mutation sites. The mimic NC or miR‐378a‐3p mimic, or inhibitor NC or miR‐378a‐3p inhibitor with WT‐PDIA4 or MT‐PDIA4 were transfected or cotransfected into HEK‐293T cells. D, The luciferase activity was detected by dual‐luciferase reporter gene assay. E and F, RIP assay further examined the targeting relationship between miR‐378a‐3p and PDIA4 in OVCAR3 and SKOV3 cells. All data were expressed as mean ± *SD* (*n* = 3). *p < .05, **p < .01, ***p < .001. mRNA, messenger RNA; RT‐qPCR, reverse transcription‐quantitative polymerase chain reaction

As we mentioned above, the expression of miR‐378a‐3p was negatively related to that of PDIA4. In addition, miR‐378a‐3p was found to promote cell invasion and migration, while PDIA4 exhibited the opposite tendency. Based on this, we speculated that there may be a connection between miR‐378a‐3p and PDIA4. miR‐378a‐3p inhibitor increased PDIA4 expression, while miR‐378a‐3p mimic reduced PDIA4 expression (Figure 4A,B, *p* < .01). The prediction through online Starbase software indicated the targeting relationship between miR‐378a‐3p and PDIA4 (Figure 4C). For the purpose of confirming the targeting relationship between miR‐378a‐3p and PDIA4, we constructed a WT PDIA4 luciferase promoter plasmid (named WT‐PDIA4) and a mutant PDIA4 luciferase promoter plasmid (named MT‐PDIA4). Results from luciferase activity assay suggested that the transfection of WT‐PDIA4 and miR‐378a‐3p mimic decreased luciferase activity (*p* < .01), the transfection of WT‐PDIA4 and miR‐378a‐3p inhibitor increased luciferase activity (*p* < .01), but the transfection of MT‐PDIA4 and miR‐378a‐3p mimic or miR‐378a‐3p inhibitor exhibited no change in luciferase activity (Figure 4D). Subsequently, we further confirmed the targeting relationship between miR‐378a‐3p and PDIA4 in OVCAR3 and SKOV3 cells through RIP experiments, which revealed that miR‐378a‐3p was increased through PDIA4 antibody treatment (Figure 4E,F, *p* < .01). Meanwhile, a large amount of PDIA4 protein was detected in the RNA‐protein conjugate by Western blot assay (Figure 4E,F). These results elucidate that PDIA4 directly binds to miR‐378a‐3p.

### 3.5 miR‐378a‐3p promotes ovarian cancer cell growth by suppressing PDIA4 expression

**Figure 5:**
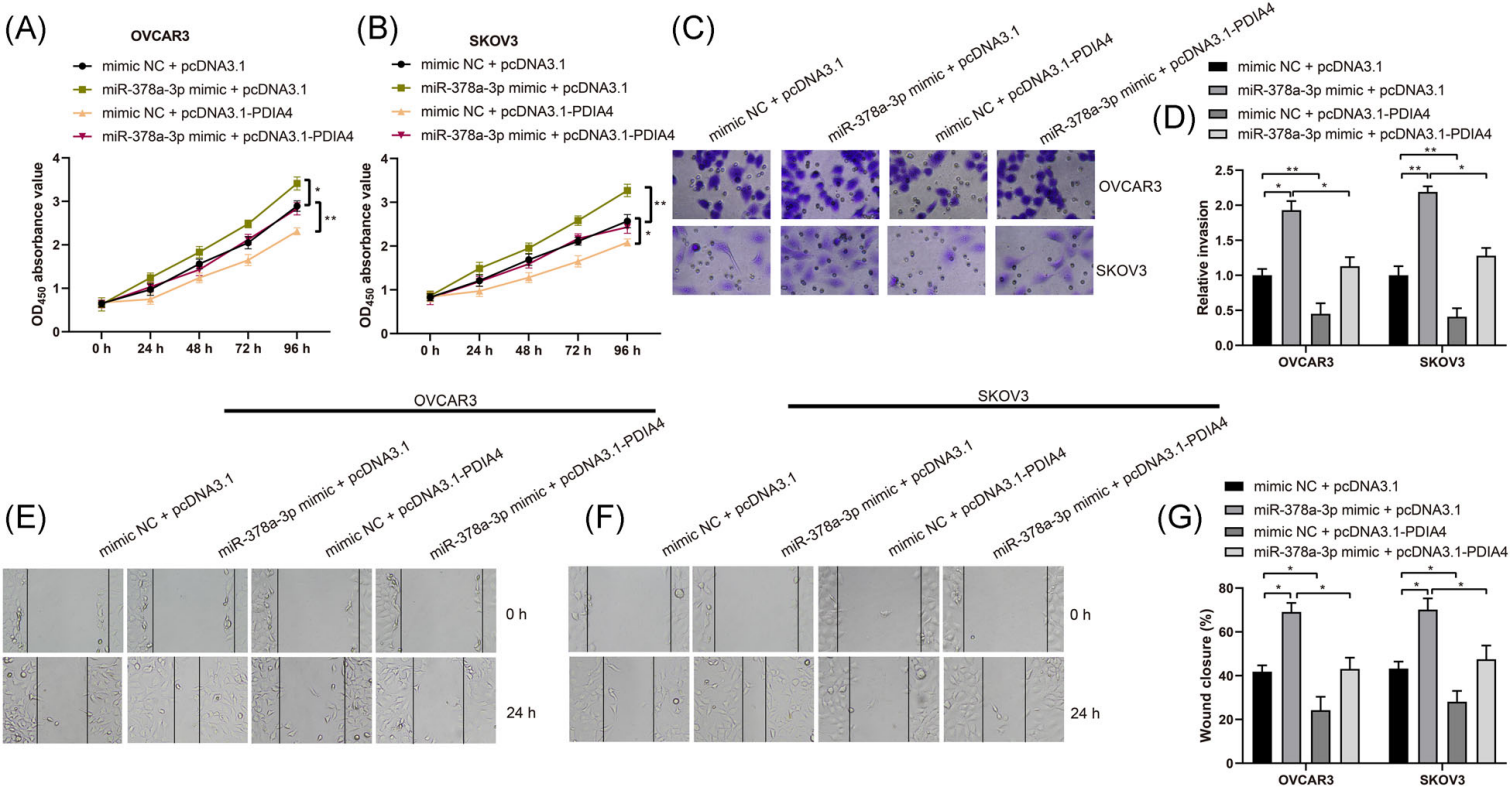
miR‐378a‐3p contributes to ovarian cancer cell growth by suppressing PDIA4 expression. Ovarian cancer cells OVCAR3 and SKOV3 were transfected withpcDNA3.1, pcDNA3.1‐PDIA4, mimic NC, or miR‐378a‐3p mimic, respectively. A and B, Viability of OVCAR3 and SKOV3 cells was measured by MTT assay. C and D, Invasion ability of OVCAR3 and SKOV3 cells was detected by Transwell assay. E–G, Migration ability of OVCAR3 and SKOV3 cells was detected by scratch test. All data were expressed as mean ± *SD* (*n* = 3). *p < .05, **p < .01. MTT, 3‐(4,5‐dimethylthiazol‐2‐yl)‐2,5 diphenyltetrazolium bromide

The next step was to probe into whether miR‐378a‐3p regulated PDIA4 expression to modulate ovarian cancer cell progression. The malignant phenotypes of OVCAR3 and SKOV3 were heightened in comparison of the mimic NC + pcDNA3.1 group and the miR‐378a‐3p mimic + pcDNA3.1 group. On the contrary, OVCAR3 and SKOV3 cell proliferation, migration, and invasion abilities were weakened in comparison of the mimic NC + pcDNA3.1 group and the mimic NC + pcDNA3.1‐PDIA4 group, together with the miR‐378a‐3p mimic + pcDNA3.1 group and the miR‐378a‐3p mimic + pcDNA3.1‐PDIA4 group (Figure 5A–G, all *p* < .01). It is concluded that miR‐378a‐3p contributes to promote the growth of ovarian cancer cells by restricting PDIA4 expression.

### 3.6 miR‐378a‐3p functions in ovarian cancer by activating PI3K/AKT signaling pathway

**Figure 6:**
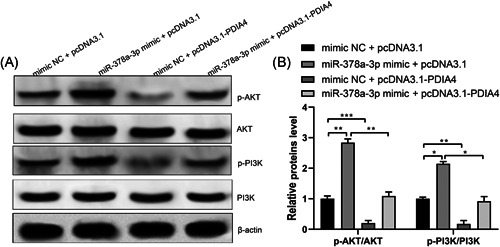
miR‐378a‐3p functions in ovarian cancer growth through the activation of the PI3K/AKT signaling pathway. A and B, Ovarian cancer cells SKOV3 were transfected withpcDNA3.1, pcDNA3.1‐PDIA4, mimic NC, or miR‐378a‐3p mimic, respectively. Total AKT and PI3K protein levels and their phosphorylation levels were determined by Western blot assay. All data were expressed as mean ± *SD* (*n* = 3). *p < .05, **p < .01, ***p < .001. PI3K/AKT, phosphatidylinositol‐3 kinase/serine/threonine kinase

Further investigation focused on finding out the downstream signaling pathway of miR‐378a‐3p and PDIA4 in ovarian cancer, we found that miR‐378a‐3p mimic and pcDNA3.1‐PDIA4 had no effect on AKT and PI3K levels, while miR‐378a‐3p mimic enhanced the expression levels of p‐AKT and p‐PI3K, and pcDNA3.1‐PDIA4 suppressed the expression levels of p‐AKT and p‐PI3K. Moreover, pcDNA3.1‐PDIA4 was found to largely eliminate the promoted effect of miR‐378a‐3p mimic on the expression of p‐AKT and p‐PI3K (Figure 6A,B, all *p* < .01). These results imply that miR‐378a‐3p activates PI3K/AKT pathway by regulating PDIA4, thereby playing a role in promoting the malignant phenotypes of ovarian cancer cells.

## 4 DISCUSSION

Despite optimal treatment, the 5‐year survival for ovarian cancer is about 30% and the majority of patients succumb to their disease.^24^ The varying expressions in miRNAs have been identified in many cancer types including ovarian cancer. The understanding of the role of miRNA in ovarian cancer will provide essential insight into the diagnosis and treatment of this disease.^25^ Nevertheless, the inner molecular mechanisms of how miR‐378a‐3p functions in ovarian function remain largely unknown. In this current study, we discovered that miR‐378a‐3p promoted ovarian cancer cell growth via negatively modulating PDIA4 and PI3K/AKT pathway.

Since miR‐378a‐3p has been revealed to be elevated in ovarian cancer cells,^5^ we first detected the expression of miR‐378a‐3p in ovarian cancer, and the findings indicated that miR‐378a‐3p was elevated in ovarian cancer, which was in line with the aforementioned trend. Except that, the oncogenic role of miR‐378a‐3p has also been investigated in other human cancers, Redova et al.^26^ have found that miR‐378 expression is upregulated in renal cell carcinoma. Another study has indicated that miR‐378 is elevated in cholangiocarcinoma tissues and cells in comparison with adjacent normal tissues and HIBEC cells.^27^ In addition, we further investigated the functions of miR‐378a‐3p in malignant phenotypes of ovarian cancer cells. The results suggested that miR‐378a‐3p acted as an inducer in ovarian cancer development. A prior study has indicated that miR‐378 provides significant insights into the inner mechanisms underlying the modulation of ovarian estradiol production through targeting aromatase in granulosa cells.^28^ Another study has demonstrated that miR‐378 expression is enhanced in ovarian cancer specimens versus the normal ovarian surface epithelial cells. In addition, miR‐378 together with its downstream targets has been considered as an important indicator for the response to antiangiogenic therapy.^12^ Except for the oncogenic role in cancers, the tumor‐suppressive function of miR‐378a‐3p has also been demonstrated in some articles. Ikeda et al.^29^ have supported that miR‐378a‐3p expression is decreased in breast cancer cells and tissues, and low miR‐378a‐3p expression presented with poor prognosis patients with breast cancer.^29^ Ding et al.^10^ have stated that miR‑378a‑3p may be considered as a tumor suppressor in esophageal squamous cell carcinoma cells via targeting Rab10. These data imply that the role of miR‐378a‐3p might vary in different types of cancers. More importantly, in a study, Xu et al.^30^ have identified that miR‐378a‐3p could be a tumor inhibitor to sensitize ovarian cancer cells to cisplatin by targeting MAPK1/GRB2.^30^ The contradictory results of Xu's article from ours might be caused by the different sample size and other uncertainties during the experiment, and our findings should be verified in our future research.

Next, the correlation analysis showed that miR‐378a‐3p was negatively correlated with PDIA4. Also, based on the results of luciferase activity and RIP assays, we found that miR‐378a‐3p could combine with PDIA4. Mechanism investigations of an article have suggested that MAPK1 and GRB2 are found to be the targets of miR‐378a‐3p, and upregulated miR‐378a‐3p is able to increase the cisplatin sensitivity of ovarian cancer cells via binding to MAPK1 and GRB2,^13^ implying that miR‐378a‐3p functions in ovarian cancer by targeting other genes. Further investigation is warranted to confirm the relationship between miR‐378a‐3p and PDIA4. It is suggested in a study that DIA4 was downregulated in drug‐resistant ovarian cancer tissues relative to the sensitive ones, and inhibited PDIA4 predicted shorter disease‐free survival and overall survival in both the tested surgical specimens and the TCGA cohort.^17^ Some researchers also concentrated on the roles of PDIA4 in other human cancers. For example, Kuo et al.^31^ have proposed that PDIA4 is of importance in the growth and death of human cancer cells, which is a potent therapeutic target for tumor.^31^

Furthermore, we wanted to elucidate through which signaling pathway that miR‐378a‐3p and PDIA4 played a role in ovarian cancer, and corresponding findings suggested that miR‐378a‐3p stimulated ovarian cancer cell growth by downregulating PDIA4 and activating PI3K/AKT pathway. The signaling pathways related to the invasion and migration of ovarian cancer cells include GSK/3β, Wnt/β‐catenin, MEK/ERK, and PI3K/AKT pathways. In this study, we found that through experimental screening, miR‐378a‐3p regulated the PI3K/AKT signaling pathway through targeting PDIA4. It is reported that PI3K/AKT signaling pathway plays a role in ovarian cancer growth,^19^, ^20^ which can explain that miR‐378a‐3p performs biological functions through the modulation of the PI3K/AKT signaling pathway. Evidence has revealed that the PI3K/AKT pathway is indispensable for regulating some cellular and molecular functions.^32^ Research has indicated that the activation of the PI3K/AKT pathway is important for ovarian cancer tumorigenesis and in chemotherapy resistance.^33^ The PI3K/AKT pathway is found to induce cancer cell proliferation and survival, and AKT is highly expressed in epithelial ovarian cancer.^34^ Another study has suggested that overexpressed miR‐378 attenuates high glucose‐restricted osteogenic differentiation via binding to CASP3 and activating the PI3K/AKT pathway.^1^ However, the functions of miR‐378a‐3p, PDIA4, and PI3K/AKT pathway in ovarian cancer progression need further confirmation.

In conclusion, this study highlights that miR‐378a‐3p plays an oncogenic role in ovarian cancer cell development through modulation of the PDIA4‐mediated PI3K/AKT pathway. This helps us to understand the pathomechanism and development of ovarian cancer from the gene level, and provides a new direction for targeted therapy of ovarian cancer.

## CONFLICT OF INTERESTS

The authors declare that there are no conflict of interests.

## AUTHOR CONTRIBUTIONS

Yao Chanjiao and Chen Chunyan contributed to the conception of the study; QiuXiaoxin and Han Youjian contributed significantly to analysis and manuscript preparation; Yao Chanjiao, Chen Chunyan, and QiuXiaoxin performed the data analyses and wrote the manuscript; all authors helped perform the analysis with constructive discussions; QiuXiaoxin and Han Youjian supervised and checked the manuscript.
